# Static and dynamic methods to determine adsorption isotherms of hemp seed (*Cannabis sativa* L.) with different percentages of dockage

**DOI:** 10.1002/fsn3.744

**Published:** 2018-07-20

**Authors:** Fuji Jian, Darsana Divagar, Jennifer Mhaiki, Digvir S. Jayas, Paul G. Fields, Noel D. G. White

**Affiliations:** ^1^ Department of Biosystems Engineering University of Manitoba Winnipeg MB Canada; ^2^ Morden Research and Development Centre, Agriculture and Agri‐Food Canada c/o Department of Biosystems Engineering University of Manitoba Winnipeg MB Canada

**Keywords:** adsorption isotherms, dockage, mathematical model, static and dynamic method, thin‐layer drying

## Abstract

Adsorption and desorption isotherms of hemp seeds with 0%, 5%, 10%, 15%, and 20% of dockage were determined using the salt solution static (SSS) method. The wet hemp seeds with 0% dockage were also dried at 30℃ with 50% RH, 35℃ with 30% and 50% RH, and 40℃ with 30% and 50% RH inside a thin‐layer dryer (thin‐layer dynamic method). The hemp seeds with different percentages of dockage showed hysteresis, and this hysteresis became more obvious with the decrease of temperature. At the same condition, the equilibrium moisture content of hemp seeds with 0% dockage was approximately 0.5 percent points lower than that of the hemp seeds with dockage. The best equation to fit the equilibrium moisture content data under constant temperature and RH was the modified GAB equation for both adsorption and desorption isotherms. The constant rate period of drying was observed for <0.75 hr when drying air RH was 30% or when drying air temperature was 40℃. The Henderson and Pabis model was the best model to fit the thin‐layer drying data. The equilibrium moisture contents measured by the SSS method were lower than those measured by the thin‐layer dynamic method when temperature was ≤35℃.

## 1 INTRODUCTION

Commercial production of industrial hemp (*Cannabis sativa* L.) has been permitted in Canada since 1998 and Canada produced more than 27,000 tonnes of hemp seeds in 2016. In Canada, hemp seeds are harvested when about 70% of the seeds are ripe and the average moisture content of the harvested seeds is about 16% to 27% (dry basis used in this article). The safe storage moisture content of hemp seeds is about 10% (unpublished data). The harvested seeds contain up to 15% dockage including green leaves, buds, stems, weed seeds, and other negligible amounts of foreign and fine materials, and this dockage is mostly not cleaned out while the seeds are stored for up to 1 year. The hemp seeds with dockage at high moisture conditions have the potential to spoil easier than other stored major crop seeds, as well high bacterial contamination. Every year, about 5% of seeds are rejected due to the mold and heat damage or bacterial and fungal contamination (personal communication with Agronomists from Manitoba Harvest Fresh Hemp Foods, Canada). The unit price of hemp seeds is approximately more than six times that of wheat. Therefore, spoilage in a single bin will result in a huge financial loss for hemp producers.

To reach the required safe storage moisture content, hemp farmers usually ventilate to cool and/or dry the stored seeds. To determine the ideal drying and storage condition such as drying air temperature and relative humidity (RH), both desorption and adsorption isotherms of the hemp seeds are required. Desorption and adsorption isotherms of hemp seeds have not been determined and farmers currently use the guidelines for canola because hemp seeds have approximately 30% to 35% oil content, which is close to some varieties of canola. However, high oil canola varieties usually have more than 42% oil content (Sun, Jian, Jayas, White, & Fields, 2014). The other chemical components such as protein inside hemp seeds are different from those of canola. Thin‐layer drying rate and drying constants such as water diffusivity during drying have also not been determined for hemp seeds. In the literature, desorption and adsorption isotherm of other crop seeds were determined using clean seeds. It is not known whether different percentages of dockage influence the isotherms of the hemp seeds or not. Therefore, the desorption and adsorption isotherm of hemp seeds with different percentages of dockage are required by the hemp industry.

Static and dynamic methods are usually used to determine the desorption and adsorption isotherms. Both methods are to allow the tested sample to equilibrate with the ambient air under controlled constant temperature and relative humidity. During the test period, the mass of the sample is sequentially measured, so the moisture content of the sample could be determined. The static method usually uses saturated salt solutions kept inside desiccators with still air (referred to as SSS method). The main disadvantage of the SSS method is that it is time‐consuming and spoilage of the sample can occur during the measurement period, which might result in errors. The dynamic method uses air with a low flow velocity and this low velocity air will reduce the equilibrium time. Dynamic vapor sorption (DVS) is one of the dynamic methods (Atungulua, Olatundea, & Sadakab, 2018), and the results of the SSS and DVS methods were compared (Arlabosse, Rodier, Ferrasse, Chavez, & Lecomte, 2003; Bingol, Prakash, & Pan, 2012; Penner & Schmidt, 2013; Rahman & Al‐Belushi, 2006; Schmidt & Lee, 2012) using rice and processed food materials such as starch and food protein. Even though different studies had different conclusions, these studies generally found that isotherms measured by the SSS method gave different values than the DVS method. Even though the main purpose of the thin‐layer drying test (referred to as thin‐layer dynamic method) is to determine the drying constant, this method can also be used to determine isotherms of the drying materials (Erbay & Icier, 2010; Jian & Jayas, 2018a). The isotherm developed by the SSS method is usually used to estimate grain moisture contents during grain storage and drying. This isotherm is the equation showing the relationship among equilibrium relative humidity (ERH), temperature (*T*), and equilibrium moisture content (EMC) of the materials (ERH‐T‐EMC). Compared with the SSS method, the water on the surface of the materials during thin‐layer drying can be quickly removed. Therefore, the thin‐layer dynamic method reduces the time required for the measurement. However, moisture gradients inside the drying material during the thin‐layer drying period might be higher than that when the SSS method is used. This can result in different EMC at the same ERH and temperature between these two methods. Arlabosse et al. (2003) found the SSS and DVS methods would have the same isotherm equation only if the difference between the measured mean moisture content of the sample and the surface moisture content was low, which could only be achieved for very thin samples (<0.5 mm) and for relatively high moisture diffusion coefficients (higher than 10^−9^ m^2^/s). Grain seeds are usually larger than 1 mm and effective water diffusivity is lower than 10^−9^ m^2^/s. The main reason causing the difference between the SSS and DVS methods is that the DVS uses <100 times of equilibrium time than the SSS method. The thin‐layer dynamic method uses much less time than that of the DVS method. Therefore, the EMC determined by the SSS method might be different from that of the thin‐layer dynamic method under the same environmental conditions. It is important to quantify the difference because application conditions of these developed ERH‐T‐EMC relationships needs to be defined. This defined condition can guide grain storage managers to follow correct grain drying and storage practices.

The objectives of this study were to: (a) characterize desorption and adsorption isotherms of the hemp seeds using the SSS method; (b) determine the drying rates of the hemp seeds under different drying conditions; and (c) compare the isotherms of the hemp seeds determined by the SSS and thin‐layer dynamic methods.

## 2 MATERIALS AND METHODS

### 2.1 Hemp seeds with different percentages of dockage

The seeds of hemp variety (FINOLA^®^) used in this study were directly transferred to the laboratory from a field located 50 km southwest of Winnipeg after the hemp seeds were harvested by a combine. The moisture content of the hemp seeds was 26.5 ± 0.3%. The moisture content was determined by drying samples inside a convection oven at 103°C for 5 hr (Canadian Grain Commission 2016). This moisture measurement method was verified by drying the hemp seeds with different amounts of added water and dried at 103℃ with drying period of 3–15 hr with increment of 0.5 hr. The purpose of adding different amounts of water was to make small hemp seed samples with about 28, 30, 32, 34, and 36% moisture content. It was found the initial moisture content did not significantly influence the determined moisture content when the drying time was 5 hr at 103°C. Therefore, 5 hr and 103℃ was used to evaluate the moisture content in this study.

To separate the dockage from the harvested hemp seeds, the samples were separated using three dimensional vibrating screens (Sweco^®^ Vibro‐Energy^®^ Separators, Sweco, Florence, USA) with sieve openings of 5.56, 2.03, 1.65, and 0.51 mm. The hemp seeds were collected on the sieve with the opening of 1.65 mm (referred to as sieve‐cleaned hemp seeds). The dockage collected from sieve with the opening of 5.56 and 2.03 was larger than the hemp seeds, and the dockage collected from the sieve with the opening of 0.51 was smaller than the hemp seeds. This sieve‐cleaned sample was further cleaned using a dockage tester (Carter Day Dockage Tester, Carter Day International, Inc., Minneapolis, Minnesota, USA), and the amount of dockage inside the sieve‐cleaned sample was about 1.5% (by weight). The sample cleaned through the dockage tester was referred to as Wet Sample with 0% Dockage (WSD). To get wet samples with 5, 10, 15, 20, and 25% of dockage by weight, a desired amount of dockage was mixed with the WSD, for example, the wet sample with 10% dockage was produced by mixing of 10 kg of the WSD with 1.11 kg dockage. The dockage used in this study was the dockage collected during the above‐mentioned cleaning process and from an elevator when hemp seeds were loaded into an elevator bin. The size distribution of the added dockage was 45.4%, 48.4%, and 6.2%, in the size range of <2.00 mm, between 2.00 to 3.35 mm, and >3.35 mm, respectively. The dockage was mixed with the WSD using a grain mixer (Big Cat, Type B, Red Lion, Inc., Winnipeg, Manitoba, Canada) for 0.5 hr. About half of these wet samples with 0, 5, 10, 15, and 20% of dockage were dried to about 5% moisture content (referred to as dry sample) by placing about 5‐mm thickness of hemp seeds on a table at room conditions (22 to 28°C and 30 to 45% RH) for 2 weeks. These samples were referred to as dry samples with 0, 5, 10, 15, and 20% of dockage. These wet and dry samples with 0, 1, 10, 15, 20, and 25% dockage were used for this study. All these prepared samples were kept inside double layer plastic bags and stored at 5 ± 1°C for at least 10 days before use.

### 2.2 Desorption and adsorption isotherms determined by SSS method

Experiments for determining desorption and adsorption isotherms were conducted by following the method recommended by the European Project COST 90 (Spiess & Wolf, 1983). Dry and wet samples with different percentages of dockage (0, 5, 10, 15, and 20%) were brought in equilibrium with an atmosphere generated from saturated salt solutions of CH_3_COOK, MgC_l2_, Na_2_Cr_2_O_7_, NaNO_2_, NaCl, and KNO_3_ producing about 20, 32, 50, 62, 75, and 88% relative humidity (RH) at temperatures of 10, 20, 30, 40, 50 and 60℃ (Kaymak‐Ertekin & Gedik, 2004; Uribe et al., 2011; Wexler & Hasegawa, 1954). The verification of the RH and control of the temperature was the same as reported by Jian and Jayas (2018a). The hemp seeds were sampled once a week when temperature was ≥30°C or every 2 weeks when temperature was ≤20°C and moisture contents of the triplicate samples were determined. The experiment was terminated when the moisture contents determined in three sequential measurements were statistically the same.

The American Society of Agricultural and Biological Engineers has identified Modified Henderson equation, Modified Chung‐Pfost equation, Modified Halsey equation, Modified Oswin equation, Modified Guggenheim‐Anderson‐deBoer (GAB) as best equations to describe desorption and adsorption data of different seed types (ASABE Standard 2016). Details of these equations are given in the ASABE Standard. We used these and other nonlinear equations used by Jian and Jayas (2018a), to determine the best equation for modeling the desorption and adsorption isotherms using regression. Only the data associated with the hemp seeds with 0% dockage were regressed. For example, the GAB equation is:

(1) $$M_{e}\;=\;\frac{abd\varphi }{\left(1\;-\;b\varphi )(1\;-\;b\varphi \;+\;bd\varphi \right)}$$

(2) $$d\;=\;d_{0}e^{\frac{\Delta H_{d}}{R(T-273.15)}}$$

(3) $$b\;=\;b_{0}e^{\frac{\Delta H_{b}}{R(T-273.15)}}$$

where a is the monolayer moisture content (%); $\Delta H_{d}$ and $\Delta H_{b}$ are functions describing the heat of adsorption and condensation of the water vapor (J/mol), respectively; R is the gas constant (8.314 J mol^−1^ K^−1^); *T* is the temperature of the seeds(K); φ is the relative humidity (%); and *M*_*e*_ is the equilibrium moisture content (%). The modified GAB equation was further modified, and different equations were tried to modify each parameter, so the relationship among the temperature, equilibrium moisture content, equilibrium RH, and heat of adsorption and condensation was incorporated into one equation. The best‐fitted equation was the equation with the highest coefficient of determination (*R* ^2^) and smallest mean squared error (MSE) between the predicted and measured equilibrium moisture contents.

### 2.3 Thin‐layer drying

The wet samples with 0% dockage of the hemp seeds were dried using a thin‐layer dryer. The thin‐layer dryer and procedure of the drying was the same as that described by Jian and Jayas (2018a). The drying conditions were 30℃ with 50% RH, 35℃ with 30% and 50% RH, and 40℃ with 30% and 50% RH. These combinations partially represented the conditions of natural air‐drying with a heater in Manitoba, Canada. The air velocity during the entire drying period was fixed at 0.2 m/s. Six perforated trays with dimension of 20.8 × 20.8 cm^2^ were located at the centre of the drying chamber. Four *T*‐type thermocouples were installed under each tray to measure the drying air temperature, and these thermocouples were connected with a data acquisition system (34970A, Agilent Technologies Inc., Santa Clara, CA). Prior to each test, the thin‐layer dryer was run for at least 12 hr to stabilize the system. About 200 g of samples was placed on each sample tray at the beginning of the test. The mass of the trays with samples was measured every 15 min by taking out the trays. The trays were slotted back in <10 s. The drying tests were terminated when the mass of the sample determined in three sequential measurements did not change. The moisture content of the samples was measured at the beginning and the end of the drying tests by drying triplicate samples (about 10 g each) at 103℃ for 5 hr. The moisture content measured at the end of the drying was assigned as the equilibrium moisture content of the thin‐layer dynamic method. There were six replicates for each drying condition.

Drying curves were plotted as drying rate against the moisture content of the hemp seeds and the drying time. The drying rate was calculated as:

(4) $${\left(\frac{\text{dMC}}{dt}\right)}_{n}\;=\;\left(\frac{{\text{MC}}_{n-1}-{\text{MC}}_{n}}{t_{n}-t_{n-1}}\;+\;\frac{{\text{MC}}_{n}-{\text{MC}}_{n+1}}{t_{n+1}-t_{n}}\right)/2$$

where (dMC/d*t*)_*n*_ = drying rate (kg kg^−1^ hr^−1^) at time *n*;* t*_*n*_, *t*_*n*−1_ and *t*_*n*+1_ = drying times at *n*,* n* − 1, and *n* + 1, respectively; MC_*n*_, MC_*n*−1_, MC_*n*+1_ = moisture contents at time *n*,* n *− 1, and *n* + 1, respectively.

### 2.4 Semi‐theoretical and empirical models of the thin‐layer drying

The method developed by Jian and Jayas (2018a) was used to model the thin‐layer drying of hemp seeds. To find the drying constant (*k*_th_), which could be used to estimate the effective water diffusivity, linear regression between time and the measured moisture contents at each tested drying condition was conducted by fitting the following semi‐theoretical model (Henderson and Pabis equation in natural logarithm format):

(5) $$\text{ln MR}\;=\;\ln\;\;A_{1}-k_{\mathrm{th}}t,\;\;\text{MR}\;=\;\frac{M_{t}-M_{e}}{M_{i}-M_{e}}$$

where *t* is the time (hr), *A*_1_ is the geometric constant, *M*_*t*_ is the grain moisture content (%) at time *t, M*_*e*_ is the equilibrium moisture content at the drying condition (%), *M*_*i*_ is the initial moisture content (%), and *k*_th_ is the drying constant (hr^−1^) which could be used to theoretically calculate the effective water diffusivity. To calculate the effective water diffusivity, infinite slab was assumed because the hemp seeds could only be dried at its top and bottom surfaces when the hemp seeds touched each other in one kernel deep thin‐layer during drying. The moisture content at the end of each thin‐layer drying condition was assigned to *M*_*e*_.

The measured data at each drying condition were also fitted to the following empirical models: Lewis (Newton), Henderson and Pabis, Modified Henderson and Pabis, Page, Modified Page, Modified Page II, Logarithmic, Two term, Two term exponential, Verma et al., and Hii‐ et al. Details of these models are given in Jian and Jayas (2018a). The drying constant (*k*) in each empirical model was found by conducting regression between the time and the measured moisture contents. For example, Equation (6) is the Henderson and Pabis model.

(6) $$\text{MR}\;=\;ae^{-kt}$$

where *k* is the regressed drying constant (hr^−1^). Four steps were used to find the best‐fitted equations of the thin‐layer drying data. The values of *R* ^2^ and MSE were the criteria used in the first step to select the best equation. The standard error of the *k* value was used in the second step. If the standard error was ≥the mean of the *k* or *k* < 0, this model was not selected. To determine whether there was significant difference between the *k*_th_ and *k* values, Student's *t* test was conducted and the model with most of no significant differences between *k* and *k*_th_ was selected. If there was no model having no significant difference between the *k* and *k*_th_ or there were more than one model having no significant difference, the model having the smallest difference between the *k* and *k*_th_ was selected. The reason for using this smallest difference was that: (a) the standard error of the *k*_th_ associated with drying at 40°C was smaller than 0.051 × 10^−10^ m^2^/s and this resulted in significant difference from all of the tested empirical equations; (b) there were more than six equations having no significant difference at other drying conditions; and (c) the smallest difference rule would find the empirical model with similar drying constant as that in the semi‐theoretical model.

### 2.5 Data analysis

To check whether the dockage influences the adsorption and desorption isotherm, two‐factorial tests were conducted at each constant temperature. The two factors were the percentages of dockage and different levels of RH. Tukey's tests were conducted to compare the moisture contents of the hemp seeds with 5, 10, 15, 20, and 25% dockage at the same temperature and RH.

To find whether there is significant difference between the moisture content predicted by the developed desorption isotherm equation and the moisture content measured by the thin‐layer dynamic method, Student's *t* test was conducted. The predicted moisture content was calculated using the best‐fitted desorption equation with considering the error of the temperature sensor located inside the drying chamber. The precision of the temperature sensor was ± 0.5°C. Therefore, the measured temperature ± 0.5°C was used to calculate the equilibrium moisture content of the hemp samples under the thin‐ layer dynamic condition.

## 3 RESULTS AND DISCUSSION

### 3.1 Desorption and adsorption isotherms determined by SSS method

**Figure 1:**
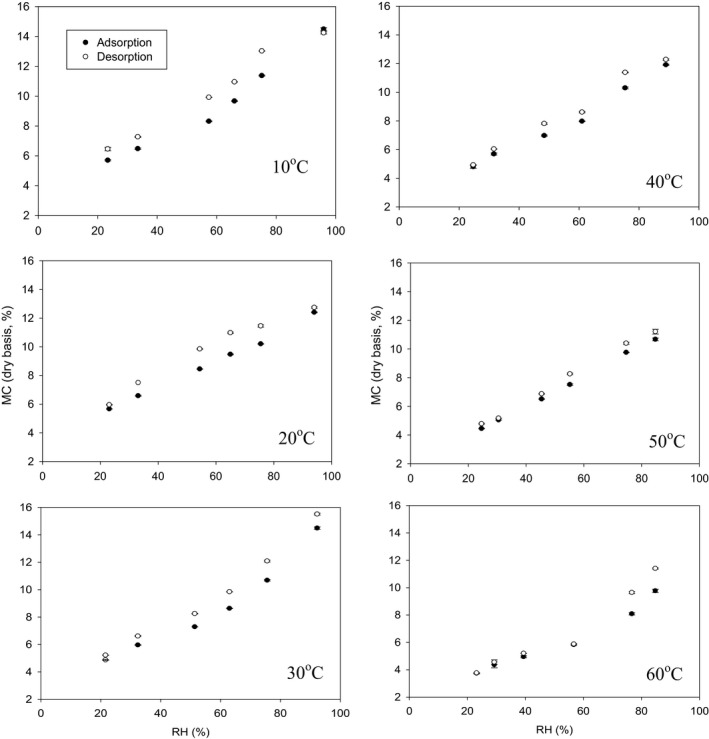
Adsorption and desorption isotherms of the dry and wet samples of hemp seeds with 0% dockage under constant temperature and RH. Error bars not shown because all standard errors were ≤0.2%

**Figure 2:**
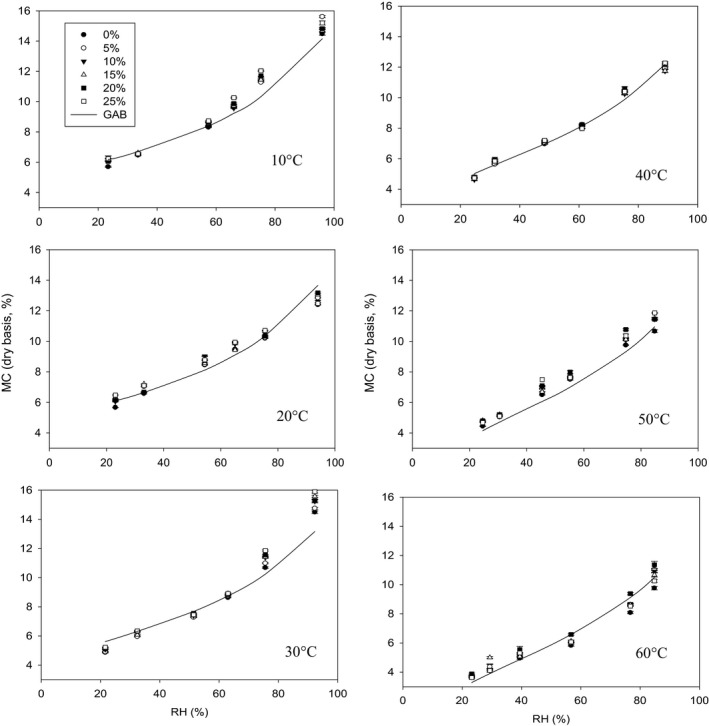
Measured and predicted moisture contents of the dry samples of hemp seeds with different percentages of dockage under adsorption conditions with constant temperature and RH. Symbols represent measured values, and lines represent predicted values using the Modified GAB model

**Figure 3:**
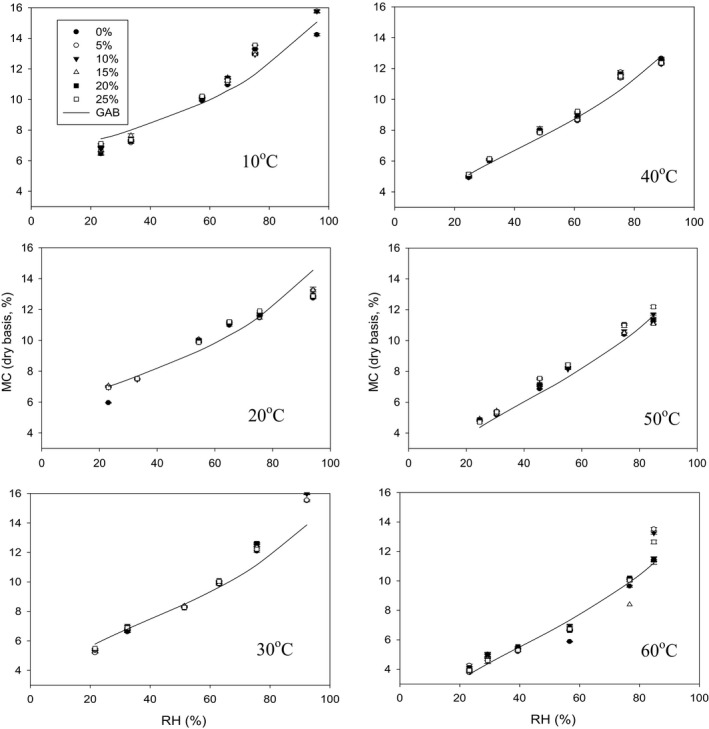
Measured and predicted moisture contents of the wet samples of hemp seeds with different percentages of dockage under desorption condition with constant temperature and RH. Symbols represent measured values, and lines represent predicted values using the Modified GAB model

The hemp seeds with different percentages of dockage showed hysteresis and this hysteresis became more obvious at lower temperatures (Figures 1, 2, 3). Hysteresis in foods is the phenomenon by which at constant water activity and temperature, a food adsorbs a smaller amount of water during adsorption than subsequent desorption (Caurie, 2007). There are significant differences in adsorption and desorption isotherms among the samples with different percentages of dockage (*p* < 0.001 for all two‐factorial tests at different temperatures). These indicated that the dockage mixed with hemp seeds significantly influenced the equilibrium moisture content of the hemp seeds. The moisture content of hemp samples with 0% dockage under both adsorption and desorption conditions was approximately 0.5 percent point lower than that of the hemp seeds with dockage (Figures 2 and 3). These result showed dockage would have a higher moisture content than hemp seeds during the storage. These higher moisture contents inside dockage might influence the safe storage of the hemp seeds. However, there were no significant differences in the equilibrium moisture content among different percentages of dockage (Tukey's test, all *p* > 0.076, Figures 2 and 3). This similar equilibrium moisture content might be caused by the large variation of the dockage at the same level percentage of dockage inside the samples because the dockage inside the initial prepared samples did not have a uniform distribution.

The best equation to fit the equilibrium moisture content data under constant temperature and RH was the modified GAB equation for both adsorption and desorption isotherms (Table 1). The parameter b in this modified GAB equation did not change with the increase in temperature. This result indicated there was no relationship between the temperature and the monolayer moisture content or the heat of condensation of the water vapor. Rahman (2005) also found the GAB model could transform into just a 3‐parameter regression model. This conclusion was the same for the adsorption and desorption isotherm of the red kidney bean (Jian & Jayas, 2018a). Basu, Shivhare, and Mujumdar (2006) also suggested that the saturated salt solution method usually does not afford sufficient information to get a complete adsorption curve. The recommended application range of the GAB model was up to 0.90 to 0.95 of relative humidity (Blahovec & Yanniotis, 2008). When adsorption has more sources (different substances aggregated in one product, etc.) and the RH is from 90% to 100%, the GAB model has a low prediction (Blahovec & Yanniotis, 2008). These conclusions were aligned with the results in our study because the biggest difference between the measured and predicted moisture contents occurred when RH > 92% (Figures 2 and 3). In this study, the lowest prediction error occurred at ≤30°C.

Most of the b values in the GAB model in the literature fall into the narrow range of 0.56 to 1.00 and depend on water activities and temperatures (Chirife, Timmermann, Iglesias, & Boquet, 1992). The values of b fall into a narrow range of 0.82 to 0.88 for protein materials, 0.65 to 0.75 for starch materials, and lower than 0.66 for oil materials (Chirife et al., 1992). In this study, the b values were from 0.55 to 0.61 (Table 1), which was consistent with the high oil content of the tested hemp seeds. Lewicki (1997) showed that the GAB model described accurately sigmoidal type isotherms when 0.24 < *b* < 1 and 5.67 ≤ *d* ≤ ∞. Keeping b and d constants in these regions fulfills the requirements of the BET model. The d value in this study was 12–72. Therefore, the isotherms of hemp seeds might be consistent with the assumption of the modified GAB model: The state of the sorbate molecules (water) in the second and higher layers of the sorbed water is equal, but different from that in the liquid‐like state. Therefore, extra energy would be required to dry the hemp seeds after liquid‐like water was removed.

### 3.2 Thin‐layer drying

**Figure 4:**
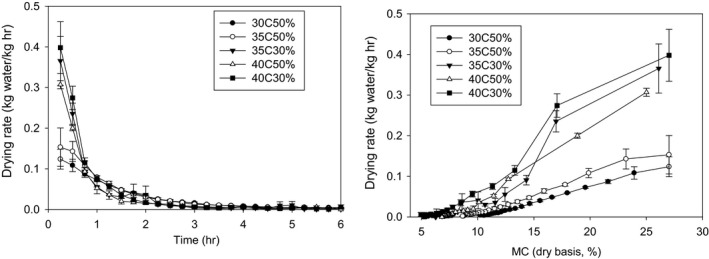
Drying rate of hemp seeds under different drying temperatures, RHs, and drying times

The constant and falling rate periods of drying of hemp seeds were observed (Figure 4). Constant rate period was observed during the initial <0.75 hr when RH was 30% or when temperature was 40°C. For the other drying conditions (30 and 35℃ with 50% RH), the constant rate period was not obvious (Figure 4). For the constant rate drying, the critical moisture content was reached in <0.75 hr and the critical moisture content was about 12%. In the falling rate period, the drying rate was the same regardless of the drying condition. The drying rate was <0.1 kg kg^−1^ hr^−1^ in the falling rate period.

### 3.3 Semi‐theoretical and empirical models

The thin‐layer drying data regressed by the Equation (5) had *R* ^2^ > 0.65 (Table 2). This indicated that: (a) the ln (MR) and the drying constant at different drying conditions followed a linear or quasi‐linear relationship; (b) the drying of the hemp seeds followed the lumped drying principle; and (c) the *k*_th_ could be used as a criterion to evaluate the best‐fitted empirical models and estimate the effective water diffusivity (Jian & Jayas, 2018a) of the hemp seeds under different drying conditions.

The values of *R* ^2^ associated with all the tested 11 empirical models were >0.9 under any drying condition (Table 2). In general, the modified Page model had the highest *R* ^2^ and lowest MSE, followed by the modified Henderson and Pabis model, then the Henderson and Pabis model. The difference between the highest and lowest *R* ^2^ at any drying condition was <0.3. The difference between the highest and lowest MSE values was close to 0. There were more than three models having the same *R* ^2^ and MSE values at any drying condition. Therefore, it was not technically possible to find the best‐fitted empirical model by only using the values of *R* ^2^ and MSE.

**Figure 5:**
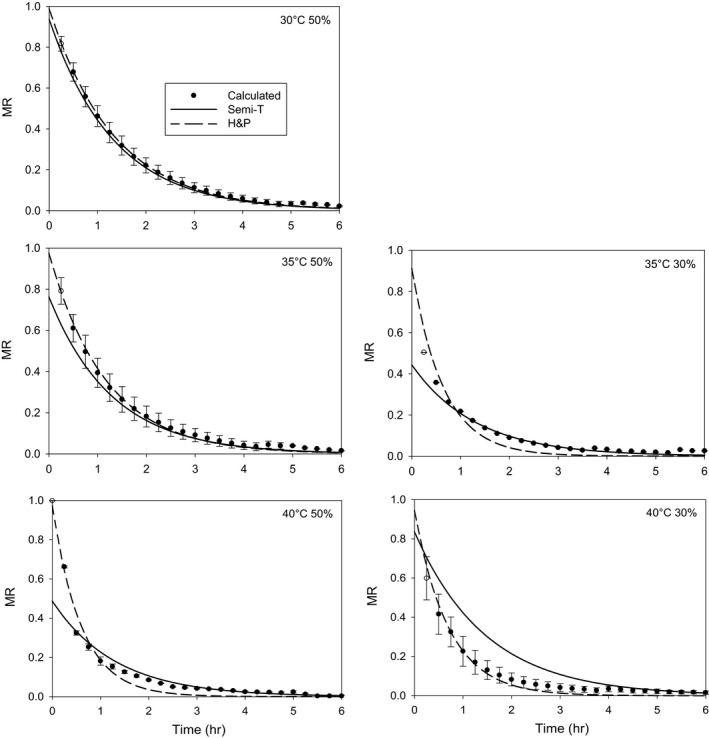
Calculated and predicted MR at different drying conditions. In the graph, Calculated = calculated MR using the measured moisture contents, Semi‐T = predicted MR using the semi‐theoretical model (Equation (5)), and H&P = predicted MR using the Henderson and Pabis model

The following models had the negative *k* value at some drying conditions: modified Henderson and Pabis, modified Page, modified Page II, Verma et al., and Hii et al. models. The standard error of the regressed *k* was more than 10 times larger than the *k* in the two term exponential and Verma et al. models. The Henderson and Pabis model had the smallest difference between the *k*_th_ and *k* in three of seven cases (Table 2). Therefore, the Henderson and Pabis model was the best model, which could be used to simulate the thin‐layer drying condition (Figure 5). This conclusion was consistent with the result reported by Jian and Jayas (2018a).

### 3.4 The moisture content determined by salt solution static and thin‐layer dynamic methods

The equilibrium moisture contents of the clean seeds predicted by the best‐fitted desorption isotherm equation were significantly lower than that measured by the thin‐layer dynamic method when temperature was ≤35°C (Table 3). Lower the temperature resulted in a much larger difference between the predicted and measured equilibrium moisture content. The maximum difference between the moisture contents measured by the thin‐layer dynamic method and that predicted by the best‐fitted desorption isotherm equation was 2.0 percentage points, while the maximum difference between the moisture contents measured by the SSS method and that predicted by the best‐fitted desorption isotherm equation at the same environmental condition was <0.1 percentage points. Therefore, the equilibrium moisture content determined by the SSS method was different from that of the thin‐layer dynamic method. There was the same trend for the red kidney beans (Jian & Jayas, 2018a). This difference might be caused by the difference of the measurement methods. It was assumed the equilibrium status between the ambient air and the seed sample was established if the moisture content did not change in the three sequential measurements regardless of the measurement method. However, the thin‐layer dynamic method used much shorter time for these three measurements (only about 1/700 to 1/1000 of the time used for the SSS method) than the SSS method used. During desorption of the samples, there was a moisture gradient between the core and surface of the seed kernels and this gradient was the main drying force (Jian & Jayas, 2018b). This gradient would be decreased with the increase in the desorption (drying) time and the temperature. If the gradient was not small enough (e.g., the difference between at the surface and the core was ≥1 percentage point), the equilibrium status might not be reached even though the moisture content in the three sequential measurements did not change. This was common phenomena during high temperature drying when rapid drying is conducted inside a high temperature dryer (Fuji Jian, unpublished data). Dalgic, Pekmez, and Belibagli (2012) found the same material dried using different drying methods could have slight difference in desorption and adsorption isotherms.

Arlabosse et al. (2003) reported that when the apparent water diffusion coefficient of the material was <10^−9^ m^2^/s, internal diffusion would be a limiting factor resulting in a difference between the SSS method and DVS methods. The estimated effective water diffusivity of the hemp seeds at any tested drying condition was <7.7 × 10^−9^ m^2^/s (Table 2). At the end of thin‐layer drying, availability of water at the surface of the seed would be the main limitation for water evaporation (Jian & Jayas, 2018b). Therefore, a difference between the SSS method and thin‐layer dynamic method could already be anticipated. Bingol et al. (2012) reported that at 0 and 98% RH for all forms of rice, there were approximately 8 to 11% and 7 to 9% differences, respectively, between DVS and SSS methods. They also discovered that this difference was higher at a water activity range of 0.40 to 0.80, and this difference would decrease when the measurement time of the DVS method was extended. Therefore, one should take caution when using the desorption and adsorption isotherm equation to predict grain moisture content in the practice of grain storage and drying.

There are commercial cables with temperature and RH sensors for use in grain bins. The measured temperature and RH are used to estimate grain moisture contents using the isotherm equations provided in the literature. This estimated moisture content can have >15% error of the true grain moisture content (Gonzales, Armstrong, & Maghirang, 2009). These isotherm equations are also used to develop mathematical models to estimate grain moisture contents during grain storage (Jian, Chelladurai, Jayas, & White, 2015), aeration, and natural air‐drying (Lopes, Neto, & Santiago, 2014). These developed models used one of the following assumptions: (a) the grain equilibrates with the intergranular air; (b) does not equilibrate; and (c) equilibrates by following a logarithmic relationship (Lopes et al., 2014). The difference on equilibrium moisture contents between the static and dynamic methods should be considered for these assumptions and model development.

## 4 CONCLUSIONS

The main objectives of this study were to characterize desorption and adsorption isotherms of the hemp seeds using SSS method, determine the drying rates of the hemp seeds under different drying conditions, and compare the equilibrium moisture content between the SSS and thin‐layer drying methods. The hemp seeds with different percentages of dockage showed hysteresis, and this hysteresis became more obvious with the decrease in temperature. There are significant differences in adsorption and desorption isotherms among the samples with different percentages of dockage. The isotherms of hemp seeds might be consistent with the assumption of the modified GAB model. The constant and falling rate periods of drying of hemp seeds were observed, and the constant rate period was <0.75 hr when RH was 30% or when temperature was 40℃. For the other drying conditions (30 and 35℃ with 50% RH), the constant rate period was not obvious. The equilibrium moisture contents predicted by the best‐fitted desorption isotherm equation, regressed using the data collected by the SSS method, were significantly lower than that measured by the thin‐layer dynamic method when temperature was ≤35℃. Lower temperatures would result in a much larger difference between the predicted and measured equilibrium moisture contents. This difference might be caused by the difference of the measurement methods because the thin‐layer dynamic method used much shorter times to determine the equilibrium moisture content than that the SSS method used. Therefore, one should take caution when using the desorption and adsorption isotherm equation to predict grain moisture content during grain drying and storage.
